# Is the Reluctance for the Implantation of Right Donor Kidneys Justified?

**DOI:** 10.1007/s00268-015-3232-0

**Published:** 2015-08-29

**Authors:** Denise M. D. Özdemir-van Brunschot, Cees J. H. M. van Laarhoven, Michel F. P. van der Jagt, Andries J. Hoitsma, Michiel C. Warlé

**Affiliations:** Division of Vascular and Transplant Surgery, Department of Surgery, Radboud University Nijmegen Medical Center, Geert Grooteplein-Zuid 10, 6525 GA Nijmegen, The Netherlands; Department of Nephrology, Radboud University Medical Center, Nijmegen, The Netherlands

## Abstract

**Background:**

The lengths of right renal veins are shorter when compared to their left counterparts. Since the implantation of kidneys with short renal veins is considered more challenging, many surgeons prefer left kidneys for transplantation. Therefore, our hypothesis is that the implantation of right kidneys from living and deceased donors is associated with more technical graft failures as compared to left kidneys.

**Methods:**

Two consecutive cohorts of adult renal allograft recipients of living (*n* = 4.372) and deceased (*n* = 5.346) donor kidneys between January 1, 2000 and January 1, 2013 were analyzed. Data were obtained from the prospectively maintained electronic database of the Dutch Organ Transplant Registry. Technical graft failure was defined as failure of the renal allograft within 10 days after renal transplantation without signs of acute rejection.

**Results:**

In the living donor kidney transplantation cohort, the implantation of right donor kidneys was associated with a higher incidence of technical graft failure (multivariate analysis *p* = 0.03). For recipients of deceased donor kidneys, the implantation of right kidneys was not significantly associated with technique-related graft failure (multivariate analysis *p* = 0.16).

**Conclusions:**

Our data show that the implantation of right kidneys from living donors is associated with a higher incidence of technique-related graft failure as compared to left kidneys.

## Introduction

The general opinion is that the implantation of renal allografts with shorter renal veins is technically more challenging. Since right kidneys have shorter renal veins, right kidneys might be associated with technique-related complications. Therefore, many surgeons prefer left kidneys for transplantation. Regarding living donor kidney transplantation, most centers prefer the selection of left kidneys.

In 1995, laparoscopic retrieval of donor kidneys was introduced by Ratner et al. [1], and nowadays, laparoscopic donor nephrectomy (LDN) has gradually become “gold standard” for kidney retrieval [2, 3]. However, laparoscopic procurement of the donor kidney also has several disadvantages as compared to the open technique, e.g., longer first warm ischemia time and operation time. Additionally, the use of the endo-vascular stapler results in loss of length of the renal vein. The right renal vein loses approximately 1.0–1.5 cm in length [4]. With regard to living donor kidney transplantation, two previously performed studies reported a possible association between the use of right living donor kidneys and venous thrombosis [5, 6], whereas other studies could not confirm this [7–14]. A recently performed systematic review with meta-analysis comparing left versus right living donor kidneys, observed a borderline significant increase in the incidence of venous thrombosis for right donor kidneys [15]. However, results from this meta-analysis should be interpreted with caution due to significant heterogeneity, and publication and selection bias.

Since renal allografts from deceased donors generally have a renal vein with a caval patch attached, the issue of right kidneys with short renal veins does, in theory, not exist in deceased donor kidney transplantation. Some studies showed a significant impact of right deceased donor kidneys on the incidence of vascular complications [16–18], whereas other studies did not [19–24].

We hypothesize that the implantation of right living donor kidneys is associated with a higher incidence of technique-related graft failures as compared to left kidneys, whereas the use of right kidneys from deceased donors does not significantly compromise technique-related outcome. To address this hypothesis, we analyzed a large consecutive cohort of kidney transplant recipients included in the Dutch Organ Transplant Registry (NOTR).

## Materials and methods

### Patients

We performed an analysis of all consecutive, living, and deceased donor, renal transplantations in adult recipients, performed between January 1, 2000 and January 1, 2014 in the Netherlands. Data were obtained from a prospectively maintained electronic database by the Dutch Organ Transplant Registry (NOTR, Dutch Transplant Foundation, Leiden, the Netherlands). The following donor and recipient characteristics and surgical parameters were extracted from the database: donor age, gender, body mass index (BMI), and donation after cardiac or brain death (DCD or DBD); recipient age, gender, BMI, smoker, diabetes mellitus, history of vascular events, previous renal transplantation(s), number of arteries, first and second warm ischemia times (respectively, WIT1 and WIT2), cold ischemia time (CIT), and center of implantation (anonymous).

### Surgery

Living donor kidneys were procured by laparoscopic and open donor nephrectomy. Initially, most centers used the open technique. During the following years, most centers gradually replaced open donor nephrectomy by laparoscopic kidney procurement. Nowadays, most centers prefer either transperitoneal LDN or hand-assisted transperitoneal LDN. In the majority of centers, left LDN is preferred over right LDN.

For deceased donors, the kidney is procured by an open approach and, when possible, a caval patch is attached.

### Outcome measures

Technical failure was the main outcome and was defined as graft failure within 10 days without signs of acute rejection. For some patients, the reason of graft failure was characterized as “primary non-function” (PNF) or “non-viable kidney” (NVK). PNF was defined as renal allograft which was good perfused but never functioned; NVK was defined as a poorly perfused renal allograft which also failed to function.

For these patients, it is difficult, if not impossible, to distinguish between technical failure or graft failure due to other factors, e.g., prolonged CIT. Therefore, separate analyses were performed: analyses excluding the patients with graft failure due to PNF or NVK and analyses including these patients.

Secondary outcomes included second warm ischemia time (WIT2) and delayed graft function (DGF). WIT2 was defined as the time from removal of the renal allograft from the ice until revascularization of the kidney; DGF was defined as the need for dialysis in the first week [25].

### Statistical analysis

Continuous variables were given as mean and standard deviation and compared using Students’ *t* test. Categorical data were given as absolute number and percentages and were compared using *χ*^2^ tests. To identify possible confounding factors, logistic regression was performed. Factors associated with kidney side (defined as *p* < 0.15) were included in the multiple regression analysis. Kaplan–Meier analysis with log-rank coefficient and Cox regression analysis was used to compare long-term graft survival. *p* values <0.05 were considered significant.

## Results and discussion

From January 1, 2000 and January 1, 2014, 9.718 consecutive renal transplantations were performed in adult recipients. In total, 4.372 renal transplantations with kidneys from living donors and 5.346 from deceased donors were performed. Demographics of donors and recipients are depicted in Tables 1 and 2. Since most centers prefer the implantation of left kidneys, for living donors, more left kidneys were procured (3097 vs 1275). For deceased donors, the number of recovered left and right kidneys was comparable (2.753 vs 2.593).

**Table 1:** Left versus right renal allograft (living donors): donor and recipient demographics and allograft functioning

	Left kidney (*n* = 3097)	Right kidney (*n* = 1275)	*p* value
Donor characteristics			
Age (year)	51.5 (SD 11.6)	50.7 (SD 12.5)	0.05
Male gender	1.375 (44.4 %)	553 (43.4 %)	0.54
BMI (kg/m^2^)	26.0 (4.1)	25.8 (SD 4.1)	0.35
Multiple arteries	83 (13.2 %)	14 (9.0 %)	0.16
Recipient			
Age (year)	48.1 (SD 13.9)	47.2 (SD 14.4)	0.04
Male gender	1.891 (61.1 %)	773 (60.6 %)	0.79
BMI (kg/m^2^)	25.1 (SD 4.1)	24.9 (SD 4.3)	0.06
Smoker	443 (17.9 %)	209 (19.7 %)	0.20
Diabetes mellitus	325 (13.2 %)	139 (13.0 %)	0.89
Vascular event	247 (9.0 %)	84 (7.4 %)	0.01
Duration dialysis (days)	473 (677)	496 (713)	0.32
Surgical parameters			
Retransplantation	358 (11.6 %)	168 (13.2 %)	0.14
WIT2 (min)	27.6 (SD 12.0)	30.1 (SD 13.6)	<0.00
CIT (min)	157 (SD 107)	162 (SD 93)	0.20
Renal outcome			
Technical failure^a^	23 (0.7 %)	21 (1.6 %)	0.01
Vascular problems	21 (0.7 %)	19 (1.5 %)	
Urological	1 (0.0 %)	0 (0.0 %)	
Other	0 (0.0 %)	2 (0.1 %)	
Technical failure^b^	26 (0.8 %)	27 (2.1 %)	<0.00
PNF	3 (0.1 %)	6 (0.5 %)	
NVK	0 (0.0 %)	0 (0.0 %)	
Creatinine			
Month 3 µmol/l	134 (SD 74)	145 (SD 116)	<0.00
Year 1 µmol/l	137 (SD 78)	135 (SD 61)	0.58
DGF	77 (2.6 %)	27 (2.1 %)	0.58

*BMI* body mass index, *CIT* cold ischemia time, *DGF* delayed graft function, *PNF* primary non-function, *NVK* non-viable kidney, *WIT2* second warm ischemia time

^a^Excluding “primary non-function” and “non-viable kidneys”

^b^Including “primary non-function” and “non-viable kidneys”

**Table 2:** Left versus right renal allograft (deceased donors): donor and recipient demographics and allograft functioning

	Left kidney (*n* = 2753)	Right kidney (*n* = 2593)	*p* value
Donor characteristics			
Age (year)	47.7 (SD 15.4)	48.4 (SD 15.4)	0.13
Male gender	1.455 (52.9 %)	1.344 (51.8 %)	0.46
BMI (kg/m^2^)	25.0 (SD 4.4)	25.1 (SD 4.4)	0.37
DCD	1051 (38.2 %)	1025 (39.5 %)	0.31
Multiple arteries	555 (20.3 %)	550 (21.3 %)	0.36
Recipient characteristics			
Age (year)	51.9 (SD 13.2)	52.5 (13.0)	0.07
Male gender	1.629 (59.2 %)	1.558 (60.1 %)	0.50
BMI (kg/m^2^)	25.5 (4.3)	25.3 (SD 4.3)	0.85
Smoker	397 (20.2 %)	406 (21.2 %)	0.46
Diabetes mellitus	425 (21.0 %)	323 (16.3 %)	<0.00
History of vascular event	297 (13.6 %)	273 (13.1 %)	0.65
Duration of dialysis (days)	1.559 (1010)	1.576 (996)	0.58
Surgical parameters			
Retransplantation	442 (16.1 %)	417 (16.1 %)	0.98
WIT1 (excluding DBD) (min)	15.8 (10.2)	16.1 (10.0)	0.51
WIT2 (min)	32.4 (13.6)	34.5 (15.2)	<0.00
CIT (min)	1071 (SD 390)	1116 (SD 387)	<0.00
Graft outcome			
Technical failure^a^	52 (1.9 %)	70 (2.7 %)	0.05
Vascular problems	44 (1.6 %)	67 (2.6 %)	
Urological	0 (0.0 %)	1 (0.0 %)	
Other	8 (.3 %)	2 (0.1 %)	
Technical failure^b^	103 (3.7 %)	130 (5.0 %)	0.02
PNF	46 (1.7 %)	49 (1.9 %)	
NVK	5 (.2 %)	11 (.4 %)	
Creatinine			
Month 3 µmol/l	170 (SD 164)	179 (SD 180)	0.07
Year 1 µmol/l	155 (SD 89)	153 (SD 87)	0.40
DGF	749 (27.3 %)	820 (31.7 %)	0.12

*BMI* body mass index, *CIT* cold ischemia time, *DGF* delayed graft function, *PNF* primary non-function, *NVK* non-viable kidney, *WIT2* second warm ischemia time

^a^Excluding “primary non-function” and “non-viable kidneys”

^b^Including “primary non-function” and “non-viable kidneys”

### Living donors

For living donors, in both uni- and multivariate analyses, the implantation of right renal allografts was significantly (*p* = 0.01 and *p* = 0.03) associated with the occurrence of technical failure (excluding NVK and PNF), Tables 1 and 3. We also observed a significant association between the implantation of right kidneys and technical failure including cases with PNF and NVK (univariate analysis *p* < 0.01 and multivariate analysis *p* = 0.01), data not shown.

**Table 3:** Univariate and multivariate analyses for technical failure (defined as excluding PNF and NVK) for right versus left renal allografts from living donors

Parameters	Univariate analysis		Multivariate analysis	
	B (95 % CI)	*p* value	B (95 % CI)	*p* value
Kidney side (right)	0.447 (0.246–0.810)	0.01	0.422 (0.197–0.902)	0.03
Donor characteristics				
Age (year)	1.016 (0.990–1.042)	0.24	–	–
Male gender	0.863 (0.476–1.563)	0.63	–	–
BMI (kg/m^2^)	1.096 (1.049–1.145)	<0.00	1.103 (1.054–1.154)	<0.00
Multiple arteries	0.997 (0.000–1.000)	1.00	–	–
Recipient characteristics				
Age (year)	0.982 (0.962–1.003)	0.09	0.976 (0.951–1.003)	0.08
Male gender	1.567 (0.865–2.839)	0.14	2.071 (0.971–4.415)	0.06
BMI (kg/m^2^)	0.996 (0.927–1.071)	0.92	–	–
Smoker	0.855 (0.355–2.059)	0.73	–	–
Diabetes mellitus	0.566 (0.173–1.847)	0.35	–	–
History of vascular event	1.961 (0.816–4.714)	0.13	1.606 (0.478–5.397)	0.44
Duration of dialysis (days)	1.000 (1.000–1.000)	0.67	–	–
Surgical parameters				
Retransplantation	0.937 (0.368–2.387)	0.89	–	–
WIT1 (min)	0.000 (0.000–1.000)	0.99	–	–
WIT2 (min)	1.032 (1.018–1.047)	<0.00	1.030 (1.012–1.049)	<0.00
CIT (min)	1.002 (1.000–1.003)	0.02	1.001 (0.998–1.004)	0.51

Separate analyses were performed including only PNF as technical failure (univariate analysis *p* < 0.01 and multivariate analysis *p* = 0.01) and including only NVK (univariate analysis *p* < 0.01 and multivariate analysis *p* = 0.03).

**Fig. 1:**
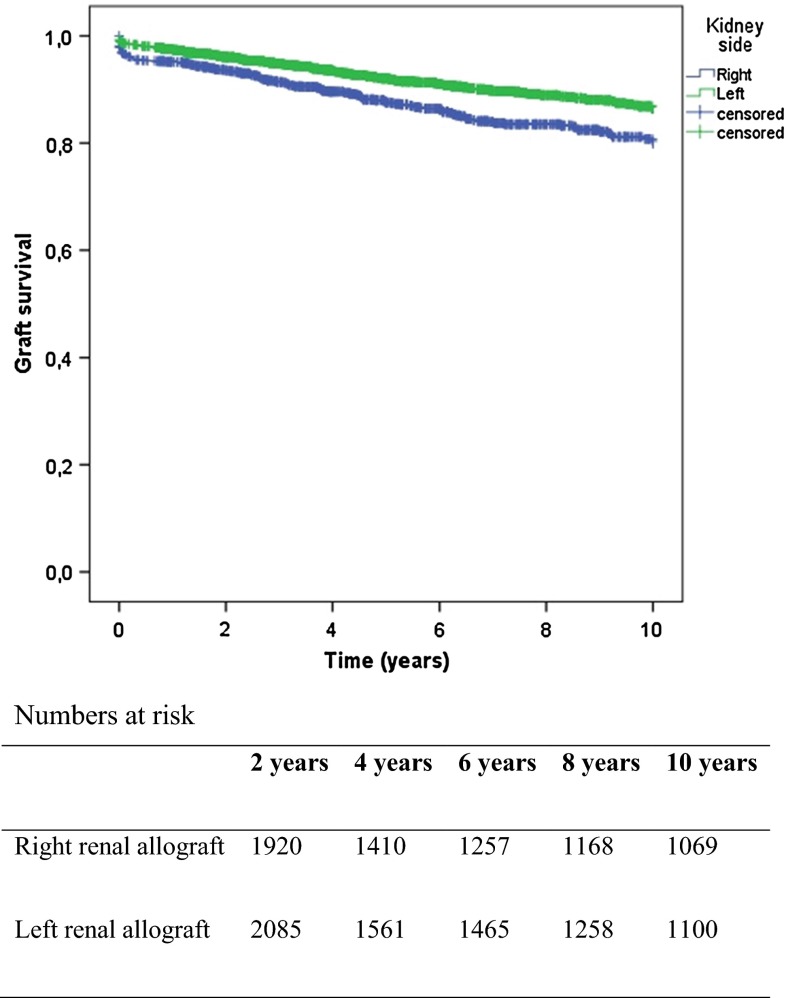
Graft survival for right versus left renal allograft, living donors (log rank 23.35 *p* < 0.000)

Right renal allografts were associated with a prolonged WIT2 (30.1 vs 27.6 min, *p* < 0.01) and a significantly higher creatinine level at 3 months (145 vs 134 micromol/l, *p* < 0.01) when compared to their left counter parts. After 1 year, creatinine levels were comparable. The implantation of right renal allografts was associated with decreased long-term (log rank 23.351 *p* < 0.01) survival for living donor kidneys, Fig. 1. Subsequently, Cox regressions analysis was performed, confirming a significant disadvantage of right renal allografts (*p* = 0.032). Analysis of data per center showed that procurement of left kidneys was preferred for living donors in most centers. In the centers procuring a relatively large proportion of right donor kidneys, the incidence of technical failure was also higher when compared to left kidneys.

### Deceased donors

For deceased donors, the incidence of technical failure, excluding PNF and NVK, was significantly increased for right sided renal allografts in univariate analysis (*p* = 0.05). However, in multivariable analysis, no association could be demonstrated (*p* = 0.16), Tables 2 and 4. When technical failure included PNF and NVK in univariate analysis, a significant association was found (*p* = 0.02) but could not be confirmed in the multivariate analysis (*p* = 0.09), data not shown. For technical failure including PNF, no significant association was found when comparing the implantation of right versus left kidney (*p* = 0.06 and *p* = 0.21); for technical failure including NVK, we only observed a significant association in the univariate analysis (*p* = 0.02 and *p* = 0.07).

**Table 4:** Univariate and multivariate analyses for technical failure (defined as excluding PNF and NVK) for right versus left renal allografts from deceased donors

Parameters	Univariate analysis		Multivariate analysis	
	B (95 % CI)	*p* value	B (95 % CI)	*p* value
Kidney side (right)	0.694 (0.483–0.997)	0.05	0.744 (0.491–1.124)	0.16
Donor characteristics				
Age (year)	0.991 (0.980–1.002)	0.10	0.994 (0.981–1.007)	0.36
Gender	0.900 (0.627–1.291)	0.57	–	–
BMI (kg/m^2^)	1.000 (0.959–1.042)	0.99	–	–
DCD	0.649 (0.453–0.930)	0.02	0.593 (0.393–0.894)	0.01
Multiple arteries	1.162 (0.757–1.783)	0.49	–	–
Recipient characteristics				
Age (year)	0.988 (0.974–1.001)	0.07	0.993 (0.977–1.009)	0.37
Male gender	0.923 (0.639–1.335)	0.67	–	–
BMI (kg/m^2^)	1.040 (.997–1.084)	0.07	1.028 (0.981–1.077)	0.25
Smoker	0.919 (0.546–1.546)	0.75	–	–
Diabetes mellitus	0.801 (0.459–1.398)	0.44	–	–
History of vascular event	0.1372 (0.807–2.333)	0.24	–	–
Duration of dialysis (days)	1.000 (1.000–1.000)	0.66	–	–
Surgical parameters				
Retransplantation	1.974 (1.315–2.964)	<0.00	2.142 (1.338–3.428)	<0.00
WIT1 (min) (excluding DBD)	1.019 (0.995–1.043)	0.16	–	–
WIT2 (min)	1.011 (1.000–1.022)	0.05	1.017 (1.006–1.028)	<0.00
CIT (min)	1.000 (1.000–1.001)	0.08	1.000 (1.000–1.001)	0.76

**Fig. 2:**
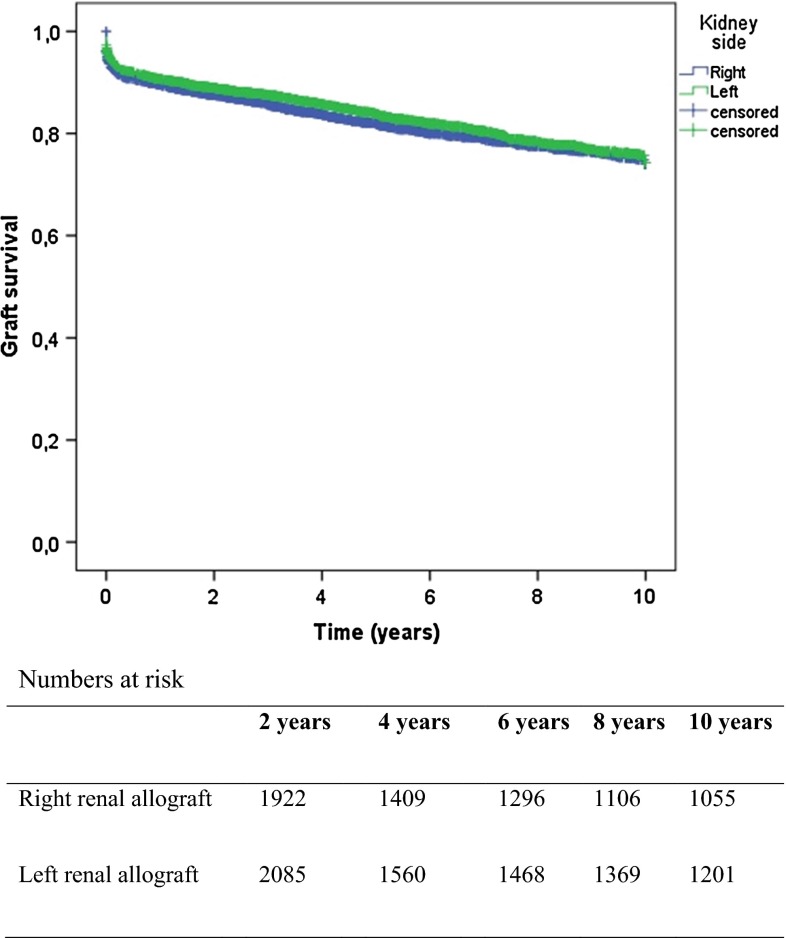
Graft survival for right versus left renal allograft, deceased donors (log rank 2.31 *p* = 0.13)

When right renal allografts were implanted, a significant longer WIT2 (34.5 vs 32.4 min, *p* < 0.01) was observed. No significant difference in post-operative creatinine was observed. No association between kidney side and graft survival was observed for kidneys from deceased donors (log rank 2.31 *p* = 0.13), Fig. 2. The use of left and right donor kidneys was equally distributed for all centers.

Our data show an association of right kidneys with the occurrence of technical failure for kidneys from living donors. The most plausible explanation is the fact that the creation of a vascular anastomosis with a short renal vein is more difficult and therefore prone to technical problems. Right kidneys from deceased donors usually have a renal vein with a caval patch. This may explain why the association between right kidneys and technical failure was not significant for deceased donor kidneys. The assumption that implantation of right kidneys is technically more challenging is underlined by the fact that the WIT2 for right kidneys was significantly longer. Another explanation could be that the right renal vein is shorter when compared to the left renal vein. The relatively short right renal vein and long renal artery can lead to compression of the renal vein in case of swelling, e.g., due to urinary obstruction or perirenal hematoma [16].

Regarding kidneys from deceased donors, there is no consensus about the influence of kidney side on graft outcome and technical failure. Some have observed a significant association of kidney side and graft survival [16–18], while others did not [22, 23, 26, 27]. Except for Vacher-Coponat et al. [18], relative small patient series are described in these studies. Vacher-Coponat et al. described adult recipients of 4900 single kidneys, procured from 2450 deceased donors in Australia and New-Zealand. A higher incidence of surgical complications and lower 1-year graft survival of right kidneys were described.

A survey among 96 transplant centers in Northern and Western Europe demonstrated that a majority of the centers preferred left-sided LDN [2]. Individual studies comparing early graft outcome for living donors have shown no significant association of right donor kidneys and venous thrombosis [7–14]. A recent systematic review and meta-analysis demonstrated a significant increased incidence of venous thrombosis, when the right kidney was used (OR 0.35; 95 % CI 0.13–0.96, *I*^2^ = 0 %) [15]. However, this disappeared after sensitivity analysis and therefore the authors concluded that the use of right kidneys did not influence the technical failure rate. This is not in line with the results from our study. A possible explanation for this discrepancy may be a type 2 error. Due to the low incidence of technical failure, approximately 0.1–8.2 % [28–32], a large cohort of renal allograft recipients are necessary to evaluate to association between technical failure and the implantation of a right renal allograft. Our cohort studied three times more patients as compared to the combined cohorts included in the meta-analysis.

Major strength of this study is that a large cohort of patients is studied and the data were obtained from a prospectively maintained database. Limitations are mainly related to the post hoc design of our study. Therefore, information regarding possible confounders, i.e., the experience of the surgeon, right or left fossa, and peri-operative hypotension, were not available. Another limitation is the fact that we could not, due to insufficient available data, analyze open and LDN separately. LDN is more likely to shorten the renal vein, and these allografts could therefore be more prone to technical difficulties during implantation.

In conclusion, our data suggest that the implantation of right deceased donor kidneys is not associated with technical failure. The implantation of right living donor kidneys is associated with an increased risk of technical failure, mainly related to the vascular anastomoses.

## Abbreviations

BMI: Body mass index
CIT: Cold ischemia time
DBD: Donation after brain death
DCD: Donation after cardiac death
LDN: Laparoscopic donor nephrectomy
WIT1: First warm ischemia time
WIT2: Second warm ischemia time
